# Comparing the delay with different anticoagulants before elective electrical cardioversion for atrial fibrillation/flutter

**DOI:** 10.1371/journal.pone.0210170

**Published:** 2019-01-03

**Authors:** Christopher Wall, Tania Jankowski, Vinci Naruka, Paula Mota

**Affiliations:** Department of Cardiology, William Harvey Hospital, Ashford, Kent, United Kingdom; Inselspital Universitatsspital Bern, SWITZERLAND

## Abstract

**Aims:**

To assess the impact of the introduction of direct oral anticoagulants upon the outcomes from elective electrical cardioversion for atrial fibrillation.

**Methods:**

This is a retrospective comparison of delay to elective cardioversion with different anticoagulants. The data was gathered from a large regional hospital from January 2013 to September 2017. There were 3 measured outcomes: 1) the time in weeks from referral to the date of attempted electrical cardioversion; 2) the proportion of patients who were successfully cardioverted; and 3) the proportion of patients who remained in sinus rhythm by the 12 week follow-up. Time-to-cardioversion was non-parametrically distributed so was analysed with Kruskal-Wallis testing and Mann-Whitney-U testing. Maintenance of sinus rhythm was analysed using z-testing.

**Results:**

1,374 patients were submitted to cardioversion. The referrals for cardioversion were either from primary care or from cardiologists. At the time of cardioversion, 789 cases were anticoagulated on warfarin (W), 215 on apixaban (A) and 370 on rivaroxaban (R). All 3 cohorts were initially compared independently using Kruskal-Wallis testing. This demonstrated a significant difference in the delay (measured in weeks) between the A and W group (A = 7, W = 9, P<0.00001); the R and W group (R = 7, W = 9, P<0.00001) and no difference between R and A (A = 7, R = 7, P = 0.92). As there was no difference between the A and R groups, they were combined to form the AR group. The AR group was compared to the W group using Mann-Whitney-U testing which demonstrated a significant delay between the groups (AR = 7, W = 9, P<0.00001). Excluding patients with prior or unknown attempts of cardioversion (n = 791), the W patients (n = 152) were less successful in achieving sinus rhythm at cardioversion than the AR (n = 431) group (W = 95% vs AR = 99% P = 0.04). However at 12 weeks, incidence of sinus rhythm was significantly different (W = 40% vs AR = 49% P = 0.049). These groups were compared by z testing. At 12 weeks' follow-up there was no statistical difference in rate of adverse consequences between the AR group and the W group, but the rate of adverse consequences was too low to draw further conclusions.

**Conclusion:**

DOACs appear to significantly shorten the latency between the decision to cardiovert and the cardioversion procedure by at least 2 weeks compared to warfarin in a real-world setting. In this study, patients who had not previously been cardioverted who were anticoagulated with warfarin had a significantly lower probability of conversion to sinus rhythm and a significantly lower probability to remain in sinus rhythm at the 12 week follow-up compared to the combined apixaban and rivaroxaban group.

## Introduction

Atrial fibrillation/flutter (AF) is the most common cardiac arrhythmia and is associated with a significant morbidity and mortality. AF is associated with increased morbidity and mortality from its adverse consequences: mainly a three-fold increased relative risk of heart failure and a five-fold increased relative risk of thromboembolic disease, particularly stroke[1]. Considering its 1.5–2% prevalence in the population and rising prevalence in the growing elderly population[1], AF is a significant and growing public health concern.

Management of AF has therefore centred on stroke avoidance and heart rate control, and its associated symptoms. Stroke avoidance is mitigated partially by anticoagulation, whilst heart rate and symptom control is mitigated with rate and rhythm controlling medications and interventions. Electrical cardioversion is used alone or in conjunction with pharmacological treatment to convert and maintain sinus rhythm in symptomatic patients[2]; this provides the additional benefit of returning the heart to physiological contraction, which is both more efficient and chronotropically autoregulating. Whilst AF has a significant long-term risk of stroke, cardioversion to sinus rhythm has an associated peri-procedural risk of thromboembolic disease of 5–7%, which is ameliorated by anticoagulation, reducing the risk to 0.8%[3]. As such, the European Society of Cardiology recommends at least 3 weeks of effective anticoagulation or use of transoesophageal echocardiography to rule out left atrial thrombus^1^ before attempting cardioversion. As transoesophageal echocardiography is labour intensive, 3 weeks of anticoagulation is commonly the preferred choice for elective cardioversion. Over the last 50 years this has been done with Vitamin K antagonists such as warfarin, but their unpredictable effects and narrow therapeutic window require regular testing and, when the INR is out of the therapeutic window, incurs cancellations and delay[4]. Direct oral anticoagulants (DOACs) such as apixaban have demonstrated equivalent safety and efficacy in post-hoc analysis of the data from Rocket-AF; whilst X-Vert and Ensure-AF have demonstrated the role of rivaroxaban and edoxaban in prospective randomised controlled trials[5–7]. Practically, these direct oral anticoagulants offer considerable potential advantages: no need for monitoring the efficacy of anticoagulation, potentially earlier cardioversion, and fewer cancellations[4,8].

This study retrospectively compares the real-world outcomes from different anticoagulants, focusing on the time delay between the decision to cardiovert and the cardioversion.

## Methods

This study retrospectively compares the time in weeks from the referral to cardioversion to the time of cardioversion in all patients for which cardioversion was attempted upon in William Harvey Hospital from January 2013 to September 2017. All patients who had attempted cardioversion were included in the data collection (n = 1,375) and all but one patient were included in the analysis (excluded because the patient was anticoagulated with dabigatran). Authors did not have access to patient identifying information, and all data was fully anonymised and de-identified, as provided in the source data.

The data was collected on a day-by-day basis at each cardioversion. As such the data was not retrospectively collected from medical records. It was gathered by the nurse managing cardioversions at the hospital that this study was based. The data was anonymised by the nurse when it was recorded, and could not be accessed by the authors writing the paper.

The date that the cardioversion department received the referral was recorded, and patients were then brought to pre-assessment clinic. If the patient had not already been on an anticoagulant before referral, they were started on either warfarin, rivaroxaban or apixaban at the pre-assessment clinic. Patients could either be referred with newly diagnosed AF, in which case they were normally not anticoagulated before referral (depending upon referrer), or a recurrence of AF where they were usually already anticoagulated.

Patients already on warfarin required an increase of target INR range from 2–3 to 2.5–3.5 in order to reduce the likelihood that the INR would drop below 2.5 at time of cardioversion. If the INR did drop below 2.5 during the weekly testing then the warfarin dose was increased and the INR was tested the following week. Once the prophylactic range was achieved these patients were booked for cardioversion at least 3 weeks afterwards. Patients who were newly started on a DOAC were booked into a cardioversion at least 3 weeks after the day they started anticoagulation. Patients who were already taking an appropriate dose of a DOAC were arranged for a cardioversion at the earliest opportunity with no minimum waiting period if they could declare that they had been taking their anticoagulant as prescribed.

The cardioversion team performed a maximum of 5 attempts at cardioversion starting at 200 Joules on the first attempt, followed by 300 Joules on the second attempt and 360 Joules for two further attempts, followed by a final attempt with 360 Joules and with the defibrillator pads in the alternate position. Failure to return sinus rhythm at the fifth attempt classified as failure at cardioversion. Patients who had been successfully cardioverted were then seen in clinic at 4 weeks and 12 weeks post-cardioversion, to observe for maintenance of sinus rhythm. This was assessed using a 12-lead ECG.

### Patients and treatment regimens

Over the course of the study period, prescribing practice changed from warfarin to DOACs. Through the course of this trial the referrer (either cardiologist or general practitioner) made the choice of anticoagulant. Some patients were refused cardioversion after referral, and these were not included in the study. The anticoagulation details and time from referral were recorded for all patients and as such no patients were excluded from the study for lack of appropriate records. Patients receiving rivaroxaban received a 20mg once daily dose (or 15mg in patients with a creatinine clearance of 30-49ml/min)[9], those receiving apixaban received 5mg twice per day (or 2.5mg twice per day if satisfying 2 of the criteria: over 80 years old; creatinine clearance less than 30ml/min; weight less than 60kg), while patients on warfarin were dosed according to INR, with an aim of 2.5–3.5. This INR was then checked on the day of cardioversion to ensure it was above 2.5, to safely proceed with cardioversion. Patients taking DOACs were not objectively monitored, but were required to sign a document stating that they had followed the anticoagulation advice. The information of any previous cardioversions was also recorded but in 494 cases (of which 488 were patients on warfarin) this status was unknown.

### Subgroup selection comparison

1,374 cases had cardioversion attempted between January 2013 and September 2017, and this was split into 789 warfarin cases, 370 rivaroxaban cases and 215 apixaban cases. These groups were compared for selection variation between the groups as shown in Table 1.

**Table 1:** Selection variation between subgroups. Data sourced from S1 File, “Subgroup analysis” tab.

Proportion of subgroup which is:	Warfarin n = 789	Rivaroxaban n = 370	Apixaban n = 215	Total n = 1,374
**Male**	72%	76%	67%	72%
**30–50 years old**	6%	7%	6%	6%
**51–60 years old**	15%	15%	14%	15%
**61–70 years old**	36%	37%	36%	36%
**71–80 years old**	32%	28%	32%	31%
**80+ years old**	11%	12%	11%	11%
**Occurring in 2013**	33%	0%	0%	19%
**Occurring in 2014**	29%	2%	0%	17%
**Occurring in 2015**	19%	28%	15%	21%
**Occurring in 2016**	11%	34%	33%	21%
**Occurring in 2017**	8%	36%	53%	23%
**1**^**st**^ **cardioversion (of those with known history)**	50%	70%	82%	66%

The table shows that the distribution of participants by age and gender is similar across all anticoagulants. However warfarin was significantly more popular in 2013 and 2014 and less popular in later years, which is unsurprising given the rise in usage of DOACs recently. As an effect of this, a greater proportion of patients in the DOAC groups had not been previously cardioverted.

As there were differences in the profiles of the patients on different anticoagulants, we statistically compared whether they were likely to contribute to any alterations in the results. Time series analysis was performed to identify any trends in delay over time.

**Fig 1:**
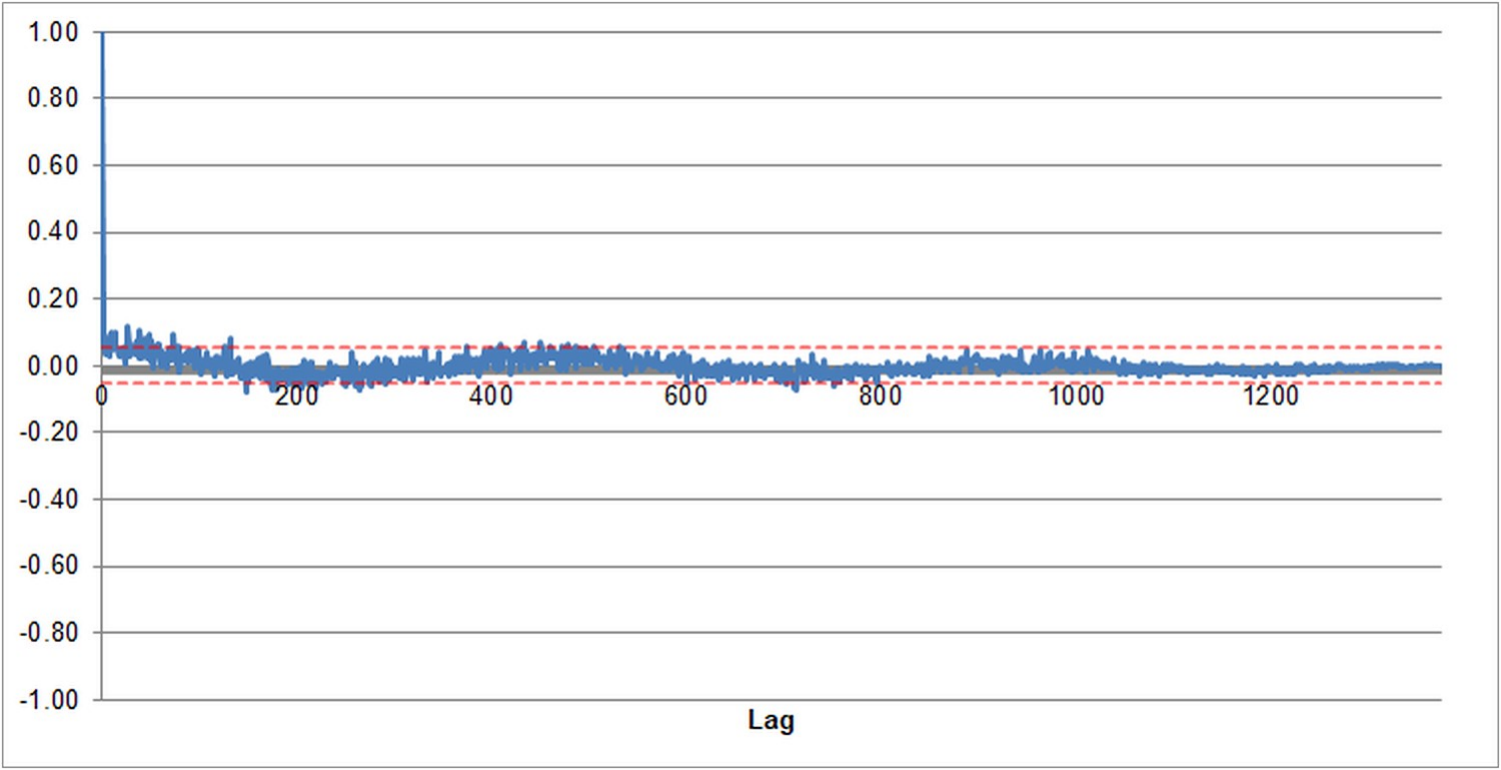
Plot of autocorrelation function over time for full dataset. Red lines show 95% significance. Data sourced from S1 File, “Full dataset” tab.

Fig 1 shows the autocorrelation function tends rapidly to zero, which demonstrates there is no significant trend which is increasing or decreasing the delay over time.

These subgroups contained a mix of patients who had newly diagnosed AF, and those who had a recurrence of AF. Chi-squared testing demonstrated that the probability density function of the delay did not fit that of a normal distribution, which is reasonable given the population taking each anticoagulant was heterogenous. Therefore the subgroups were compared using non-parametric tests: Kruskal-Wallis testing and Mann-Whitney-U testing.

## Results

A total 1,375 patients had attempted cardioversion between January 2013 and September 2017, of which 583 of these were known not to have been cardioverted before, 297 had been cardioverted before, and 495 were unknown. One patient, whose previous cardioversion status was unknown, was not included in the study because they were anticoagulated with dabigatran.

Patients who had been previously cardioverted had significantly shorter delays, as they were already on anticoagulation (although, for warfarin patients, their INR would still have to increase prior to the cardioversion taking place). As such, the data was compared both in its raw form as the primary outcome, and then weighted, to compensate for this difference, to simulate a stable population on each anticoagulant, rather than a growing or shrinking population that was occurring in DOACs or warfarin respectively.

**Fig 2:**
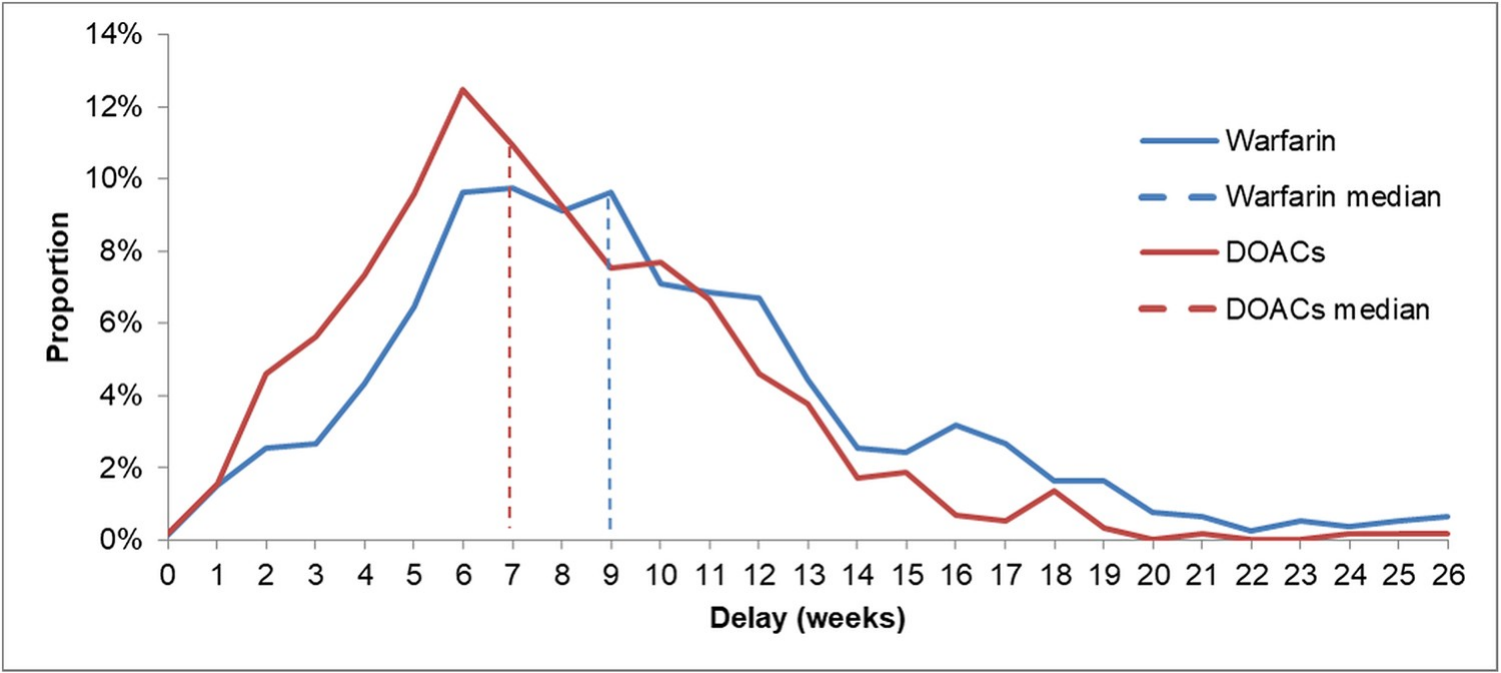
Delay in weeks between referral and cardioversion for warfarin and DOACs. Data sourced from S1 File, “Cardioversions per week graph” tab.

Fig 2 shows the average delay between referral and cardioversion for warfarin and DOACs under the primary outcome. Table 2 summarises this data for each of the anticoagulants.

**Table 2:** Median and mean delay in weeks between referral and cardioversion for different subgroups and full dataset. Weighted mean is also included for each subgroup to model stable populations. Data sourced from S1 File, “Summary—KnownNumCardioVs” tab.

	Delay in weeks						
	First cardioversion		Previously cardioverted		All		All (weighted)
	Median	Mean	Median	Mean	Median	Mean	Mean
**Warfarin**	10.0	11.1	9.0	9.9	9.0	10.5	10.7
**Rivaroxaban**	8.0	8.6	6.0	6.9	7.0	8.1	8.0
**Apixaban**	8.0	8.3	5.0	7.0	7.0	8.1	7.8
**All**	8.0	9.2	7.0	8.4	8.0	8.9	8.9

Kruskal-Wallis testing was used to show whether the differences in median delay time between subgroups were significant. This found there was no difference in median delay time between the DOACs (A = 7, R = 7, p = 0.92), but a significant difference between warfarin and rivaroxaban (R = 7, W = 9, p<0.00001) and warfarin and apixaban (A = 7, W = 9, P<0.0001). Kruskal-Wallis testing and Mann-Whitney-U testing were used to demonstrate the differences in median delay time between warfarin and the DOACs combined as one group. Both tests demonstrated a significant difference (AR = 7, W = 9, p<0.00001 in both tests).

When weighting the subgroups to simulate a stable population, rivaroxaban and apixaban showed a mean delay time which was 2.7 to 2.9 weeks shorter than the mean delay time of warfarin respectively.

For patients receiving their first cardioversion, the success rate was recorded, both immediately, and at 4 week and 12 week follow-up. Long term follow-up, beyond 12 weeks, was not recorded. A single-tailed z-test was done to determine statistical significance between the proportions in sinus rhythm for each of the anticoagulants. Apixaban and rivaroxaban were then compared to warfarin independently, and as a combined cohort. The results are recorded in Table 3.

**Table 3:** Proportion of first cardioversion patients in sinus rhythm post cardioversion, at 4 week follow-up and 12 week follow-up. Brackets contain the p-value for the z-test which compares the difference between the proportion in sinus rhythm from each treatment against warfarin. Data sourced from S1 File, “Success Rate Analysis_1stonly” tab.

	Percentage of people in sinus rhythm (p-value)		
	Immediate	4 weeks	12 weeks
**Warfarin**	95%	52%	40%
**Rivaroxaban**	99% (0.016)	53% (0,409)	47% (0.098)
**Apixaban**	98% (0.063)	56% (0.227)	51% (0.041)
**DOACs**	99% (0.011)	54% (0.309)	49% (0.049)

This table shows a significantly higher proportion of patients in the DOAC treatment arm maintaining sinus rhythm both immediately and at 12 weeks and a non-significantly higher proportion of patients in sinus rhythm at 4 weeks. This may be caused by the shorter delay to cardioversion in the DOAC arm.

The adverse events were also recorded for posterity, but events recorded at 4 weeks and 12 weeks were too few to statistically analyse and were complicated by some loss to follow-up. Of the 1,374 patients who had attempted cardioversion, 1,252 patients were seen at 4 weeks post-cardioversion and 1,137 were also seen at 12 weeks post-cardioversion.

In the warfarin group there were 7 major adverse consequences in 8 weeks (1 CVA, 1 TIA, 4 major bleeds, 1 NSTEMI). In the DOAC group there were 5 major adverse consequences (3 CVA, 1 TIA, 1 NSTEMI).

## Discussion

This study aims to demonstrate the real world challenges and outcomes in all patients who had attempted cardioversion on different anticoagulants in William Harvey Hospital. Whilst the safety and efficacy outcomes in randomised controlled trials have been thoroughly investigated, the delay incurred from requiring monitoring for warfarin has been the focus of this study. This study demonstrates a significant delay of at least 2 weeks (depending on which average used) in patients who were anticoagulated on warfarin rather than a DOAC. This additional delay at least contributes to additional poor symptomatic control for 2 weeks for patients, and is potentially responsible for the worsened efficacy of cardioversion and maintenance of sinus rhythm 12 weeks after cardioversion, observed in this study. Whilst not analysed in this study, it would also be very likely that this unpredictable response to warfarin contributes to significantly more cancellations of cardioversions[4], which may outweigh the cost difference between the anticoagulants. In addition to this, the weekly blood tests and uncertainty over date of cardioversion contribute to a well-established expense and worsened experience for patients as well as clinicians[8].

This furthers the evidence for the usage of apixaban and rivaroxaban compared to warfarin despite their relative expense.

## Supporting information

S1 File Data and calculations. This contains the original data and calculations supporting the figures and tables displayed in the manuscript.
(XLSX)
